# Different Molecular Weight Chitosan-Based Membranes for Tissue Regeneration

**DOI:** 10.3390/ma4020380

**Published:** 2011-02-10

**Authors:** Rubens M. Freitas, Rubens Spin-Neto, Luiz Carlos Spolidório, Sergio Paulo Campana-Filho, Rosemary Adriana C. Marcantonio, Elcio Marcantonio

**Affiliations:** 1 Department of Periodontology, Araraquara Dental School, Universidade Estadual Paulista, Rua Humaitá, 1680, CEP 14801-903, Araraquara, São Paulo, Brazil; E-Mails: rubensmoreno@globo.com (R.M.F.); netorubens@yahoo.com.br (R.S.-N.); adriana@foar.unesp.br (R.A.C.M.); 2 Department of Pathology, Araraquara Dental School, Universidade Estadual Paulista, 14801-903 Araraquara, São Paulo, Brazil; E-Mail: lcs@foar.unesp.br; 3 Department of Physico-Chemical (Organic Chemistry), São Carlos Chemistry Institute, University of São Paulo, 13560-970 São Carlos, São Paulo, Brazil; E-Mail: scampana@iqsc.usp.br

**Keywords:** chitosan, tissue regeneration, chitosan hydrochloride, membrane

## Abstract

Natural polymers, such as chitosan, obtained from chitin, are been widely studied for use in the tissue regeneration field. This study established a protocol to attain membranes made from this biopolymer, consisting of high or low molecular weight chitosan. The biocompatibility of these membranes was histologically evaluated, comparing them to collagen membrane surgically implanted in rat subcutaneous tissue. Fifteen *Holtzmann* rats were divided in three experimental groups: High and Low Molecular Weight Chitosan membranes (HMWC and LMWC) and Collagen membranes (C—control group); each of them with three experimental periods: 7, 15 and 30 days. As a result, after the seven days evaluation, the membranes were present and associated with a variable degree of inflammation, and after the 15 and 30 days evaluations, the membranes were absent in all groups. It is concluded that the chitosan-based membranes were successfully attained and presented comparable resorption times to collagen membranes.

## 1. Introduction

Some different techniques are being used aiming for bone formation in maxillo-facial complex defects [1], and all of them present as an essential factor, the necessity of a barrier capable of selecting the cells in the interior of these defects aiming at its regeneration. Thus, the importance of using a membrane that, besides its biocompatibility and stability, demonstrates potential to act as a cell selective agent is clear [2]. 

Researchers are showing interest in new materials, especially natural biopolymers, such as chitosan [3] that present potential in bone defect reparation, considering the limitations of some others biomaterials. 

Chitosan is a hydrophilic biopolymer obtained from the chitin partial deacetylation reaction [4], a constituent of crustaceous and insect exoskeleton, as well as the cell walls of some bacteria and fungi, and is considered the most abundant biopolymer in nature after cellulose [5]. Chitosan exhibits a variety of biological properties and, consequently, different applications for these biopolymers have been found in agriculture, industry, and recently, in the medical field as well [6,7]. Some other characteristics presented, such as its non toxicity, biocompatibility and biodegradability, allow chitosan to also be used as a film, gel, or solution [8].

The chitosan chemical structure, similar to hialuronic acid, reinforces its indication as a healing agent and repairer, due to its capacity to increase inflammatory cells’ function, such as leukocytes and macrophages, promoting cell organization and acting in the repair of ample wound healing [9].

Considering the studies already realized, the literature fail in not characterizing chitosan in the molecular weight parameter, a factor with potential importance in the biomaterial properties, as well as in the lack of studies associating its properties to function as membranes and the distinct attainment methods to allow its use in the tissue regeneration process [10,11].

The objective of this study was to develop different molecular weight chitosan-based membranes and to test the potential of these membranes for application in tissue regeneration procedures.

## 2. Experimental Methods

### 2.1. Study Design

Fifteen (15) *Holtzmann* male rats (60 days of age, weighing about 250 g) were used in all experimental groups. Totally, 45 membranes were implanted representing 3 groups: HMWC (High Molecular Weight Chitosan), LMWC (Low Molecular Weight Chitosan) and C (No crosslinked biological bovine collagen membrane (GenDerm, Baumer, Brazil)—control group) resulting in 15 membranes per group. These groups were experimentally evaluated in three periods: 7, 15 and 30 days. The materials (HMWC, LMWC and C membranes) were prepared with about 1 mm thickness and with a circular shape of 1 cm diameter. The control group membranes, according to the manufacturer, were attained from bovine cortical bone collagen.

**Figure 1:**
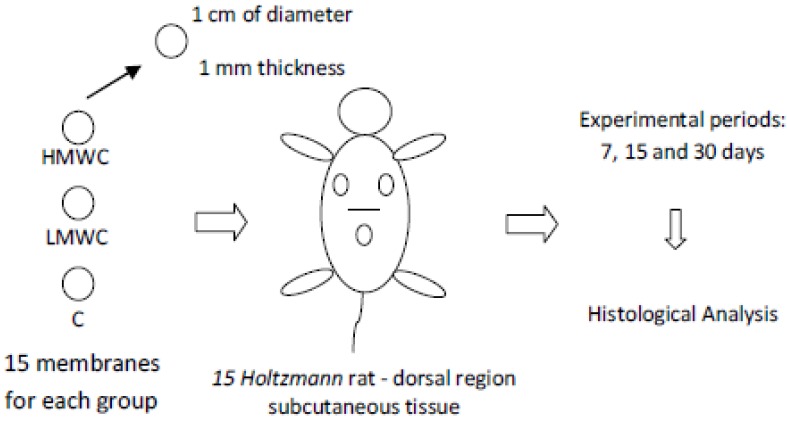
Study design schematic model.

The membranes were randomically implanted in the same animal dorsal subcutaneous tissue using a paper based raffle method. A total of 5 samples/biomaterial/period were evaluated (Figure 1).

The study protocol was approved by the Ethics in Animal Research Committee of the Araraquara Dental School (UNESP, Brazil), Process No. 24/2006, in compliance with the applicable ethical guidelines and regulations of the International Guiding Principles for Biomedical Research Involving animals (Geneva, 1985).

### 2.2. Membrane Attainment Protocol

To achieve the study objective, a method that has been previously described by our research group was used to obtain the chitosan-based gels [12]. First, shrimp crusts obtained from São Paulo State, Brazil, south coast producers were stocked in freezers and washed in running water to remove impurities, crushed in a blender, deproteinized with 1 M sodium hydroxide solution and demineralized with 0.25 M hydrochloric acid, providing the powdered chitin.

To attain chitosan, 5 g of chitin was suspended in 200 mL of a 40% 1 M NaOH solution, at 115 °C, for 6 hours and under constant stirring to promote its deacetylation, reaching a final degree of deacetylation of 85%, as measured by infrared spectroscopy (IR spectroscopy). This reaction was made in duplicate, to produce chitosan with two different molecular weights, and for that, in one of the reaction wells we introduced Sodium Borohydride (NaBH_4_) to reduce chain depolymerization, producing a high molecular weight chitosan (4 × 10^5^ kDa). In the reaction well where this substance was not used, chitosan presented a molecular weight of 9 × 10^4^ kDa. The final products were re-suspended in an acetic acid 1% solution for 24 hours, filtered and neutralized by NH_4_OH, reaction that induced the chitosan precipitation, which was washed in distilled water, filtered, and dried, and as such was available as a sponge for the membrane production. Chitosan sponges of different weights were diluted at a concentration of 20 mg/mL in 0.1 M acetic acid solution, at the same concentration as for the attainment of biomaterial gels. Two different biomaterial (gels) were produced—high molecular weight chitosan and low molecular weight chitosan; parameters were evaluated through electrophoresis, considering a medium pH of 6.0, and a stable viscosity at 37 °C. All produced chitosan showed a high purity value, also confirmed by gels electrophoresis, making it compatible to the medical use requirements.

The different biomaterials (gels) were used to produce the membranes. The procedure consisted in distributing the gels in regular Petri dishes, with 5 cm of diameter and 1 mm thickness, and afterwards placing them in a vacuum kiln at a temperature of 37 °C for 72 hours, aiming for the evaporation of the solvents. After these procedures, the membranes were sterilized with ultra-violet radiation for a period of 12 hours before *in vivo* application.

### 2.3. Surgical Procedures

The surgery was made in an aseptic environment. The animals were anesthetized by an intramuscular injection of a combination of xylazine, 0.08 mL/100 g of body weight (Xylazine Chloride—Virbaxyl 2%—Virbac do Brasil Ind. e Com. Ltda.) and ketamine, 0.04 mL/100 g of body weight (Ketamine Chloride—Francotar—Virbac do Brasil Ind. Com. Ltda.).

Afterwards, the animals were submitted to a shaving procedure of the upper dorsal region and the antiseptic process of the surgical site was made using sterile gauze embedded in an iodine solution, with the animal being placed in a ventral decubitus position over the surgical table.

One incision of approximately 10 mm was made in the medial dorsal region to allocate the chitosan-based membranes and the control membranes. The membranes were allocated in the upper dorsal subcutaneous tissue. To properly place the studied materials, the tissue was avulsed using round tip pliers, allowing the membranes to be placed in the chosen region (regions where the animals couldn`t reach using their paws), following the study design (Figure 1). The incisions were sutured with silk floss Johnson 4-0.

### 2.4. Biopsies Attainment

After the post-operative period related to the 7, 15 and 30 days, the animals were sacrificed using an overdose of 20% chloral hydrate solution. A biopsy, including the cutaneous, subcutaneous and muscular tissues involving the membranes, was obtained. These tissues (specimens) were fixed in 10% neutral buffered formalin solution for approximately 3 days, and were paraffin embedded (ethanol 70/80/95%, xylene and paraffin method). Histological micro-cuts sections of about 8 µm were made of the paraffin blocks and stained following the hematoxylin and eosin protocol.

### 2.5. Histological Analysis

Histological descriptive evaluation, done by a single, blind and expert examiner, was carried out in order to determine the characteristics of the tissues located around the tested membranes, as well as to determine the presence or not of an inflammatory reaction in the evaluated region.

## 3. Results

**Figure 2:**
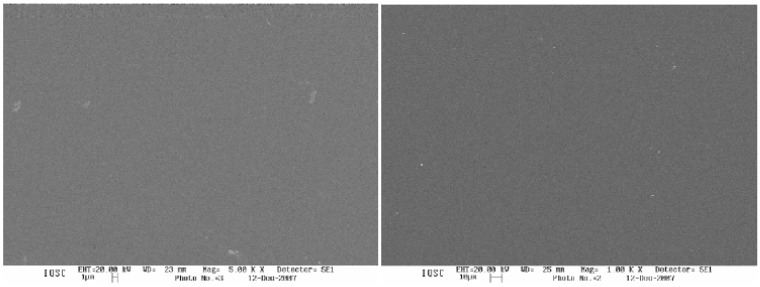
Low molecular weight chitosan (left) and high molecular weight chitosan (right) SEM (Scanning Electron Microscope) images.

The attained membranes (HMWC and LMWC) presented a homogeneous topographic surface as shown in Figure 2.

**Figure 3:**
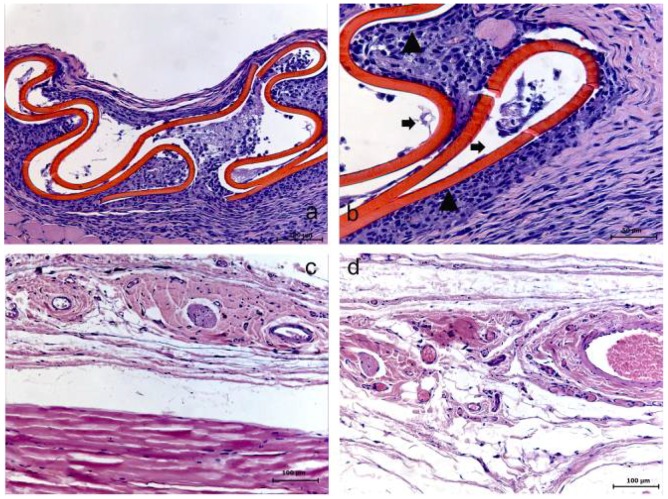
Low molecular weight chitosan (LMWC) group at 7 (**a–b**), 15 (**c**) and 30 (**d**) days. (**a**) The membrane and its surrounding tissue (HE); (**b**) Evidence of the membrane (➧) involved with the presence of a reactional tissue associated with a chronic inflammatory cell infiltrate (▲) (HE); (**c**) Absence of membrane (HE); (**d**) Tissue already organized (HE).

**Figure 4:**
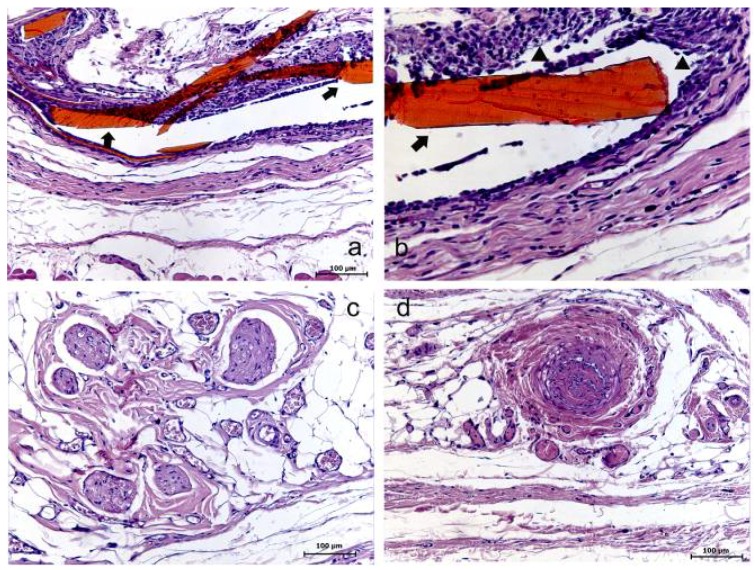
High molecular weight chitosan (HMWC) group at 7 (**a–b**), 15 (**c**) and 30 (**d**) days. (**a**) Evidence of the membrane (➧) and its surrounding (HE); (**b**) Evidence of the membrane (➧) involved with the presence of a reactional tissue associated with a chronic inflammatory cell infiltrate (▲) (HE); (**c**) Presence of collagen/fibroblastic disorganized tissue and no inflammatory reaction (HE); (**d**) Tissue presenting no evident alteration (HE).

Both HMWC and LMWC groups presented similar results, considering the membrane resorption time of 7 days. Within this period, an initial resorption process of the membrane could be evidently observed, with the presence of a reactional tissue associated with a considerable primarily chronic inflammatory cell infiltrate. Within the 15 days period, the membrane was absent in both groups, suggesting its total resorption. In this case, in the HMWC group, the region where the membrane was probably placed, presented a disorganized fibroblastic/collagen tissue and no considerable inflammatory response, and in the LMWC group, the tissue was apparently organized without inflammatory cell infiltrate. Within 30 days, in both groups, the tissue presented an apparently healthy condition in the region where the membrane was placed (Figure 3 and Figure 4).

**Figure 5:**
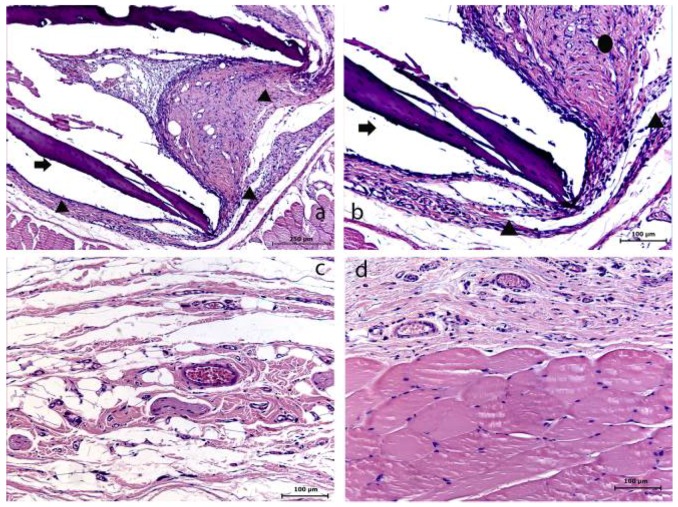
The control (C) group–collagen membrane at 7 (**a–b**), 15 (**c**) and 30 (**d**) days. (**a**) Evidence of the membrane (➧) and its surrounding (▲) (HE); (**b**) The presence of the membrane (➧) involved with a reactional tissue capsule (▲); inflammatory cell infiltrate (●) (HE); (**c**) Presence of a fibroblastic/collagen characteristic tissue (➧) and no inflammatory cell infiltrate (HE); (**d**) Tissue organized (HE).

For the Control group (C), within 7 days, the membrane was involved with a reactional tissue capsule composed of collagen fibers and fibroblastic cells apparently following the same bundle orientation, leading to a sense of granulation tissue maturity, associated with a vascular activity. After 15 days, the membrane was absent, suggesting its resorption, and the region presented tissue with collagen/fibroblastic characteristic and no apparent inflammatory activity. With 30 days, the tissue was organized (Figure 5).

## 4. Discussion

The objective of this study was to develop high and low molecular weight chitosan-based membranes and to make a histological evaluation of their application as membranes in rat subcutaneous tissue over different experimental periods, in comparison to a non crosslinked collagen membrane.

Our results suggest that chitosan-based membranes can be easily obtained, but all membranes fabricated in this study showed short resorption time, being totally present only when observed after the seven day period. The presence of the membranes was associated to a variable degree with inflammatory reaction in all cases.

The membranes studied were obtained by a process based on solvent evaporation (acid), used for the particles dissolution, and in accordance to studies such as Huang *et al*. (1999) [13] and Ghazali *et al.* (1997) [14]. This is an efficient method because the resulting membranes have good chemical stability and can be easily modified structurally, which was observed in the membranes studied.

Many studies have realized some modifications in the membranes such as: cross-linking [15,16,17], blending [18], multilayer casting and chemical modification [19]. Considering these methods, the ionic cross-linking is the most often used [15,16,17,20]. Kerakeçili *et al.* (2007) [21] used sulfuric acid anion in a study to characterize a chitosan-based membrane surface with and without ionic crosslinking, finding that the surface composition did not significantly alter, except for the sulfate interaction, and that the membrane topographic surface was more homogeneous. Some distinct studies mention other substances, such as Orrego *et al*. (2009) [22], who used glutaraldehyde and crown ether.

Rothamel *et al*. 2005 [23] evaluated the biodegradation of differently cross-linked and non-cross-linked collagen membranes in rat subcutaneous tissue, suggesting that the cross-linking of bovine collagen type I and III membranes was associated with prolonged biodegradation, decreased tissue integration and vascularization. In our study, we used a commercial non cross-linked collagen membrane, with the purpose of comparing it with the evaluated biomaterials once the chitosan-based membranes studied were non-cross-linked as well.

All the studies related above aimed only to analyze the membrane properties, not to evaluate its histological biocompatibility and potential use in the tissue regeneration process; in this regard, the present study opted to develop an attainment protocol to obtain chitosan membranes without structural modification (non cross-linked) due to the ability of some of the cross-linking agents to present some adverse effects to cells, such as carcinogenic potential, *etc*. [24], as well as the lack of “*in vivo*” studies comparing cross-linked and non-crosslinked chitosan-based membranes in the literature. Furthermore, the studies mentioned in the literature fail to characterize the membrane in relation to its molecular weight and deacetylation degree (mentioned in our methodology).

Our study, besides all the factors related to the membrane attainment protocol being well described in the methodology, provides histological results from the studied periods that were not favorable to indicate the use of the chitosan-based membrane with either molecular weight evaluated in the tissue regeneration process. The main factor, in our view, would be the presence of the membrane only in the 7 days period, associated with a variable inflammatory degree process, and the absence of these membranes in the others experimental periods. However, these results could be compared to the control group, which presented similar results, suggesting not efficient daily usage for the guided tissue regeneration field. In addition, the animal model that was used is relevant concerning our aim, and it is usually used for biomaterial biocompatibility assays, since it provides a tissue area that is highly vascularized, with easy access to the connective tissue layer and where the animal cannot interfere using its paws [25,26].

In 2006, Kuo *et al*. [11] realized a study evaluating chitosan membranes modified with NaOH gelating agent and Na_2_SO_3_ or Na_5_P_3_O_10_ cross-linking agents as barriers in a bone defect healing process created in rat’s calvaria, obtaining good results for its use in guided tissue regeneration.

Recently, Cortez *et al*. (2008) [10] demonstrated a result that differed from our study. In their preliminary study, hybrid chitosan membranes were evaluated in sheep subcutaneous tissue, where they found that even after a period of three months the membranes werestill present, and in the evaluated periods there was an intense inflammatory reaction. However, the study lacked in that they did not characterize these membranes considering the degree of deacetylation, and used a cross-linking agent based on silicate.

As the literature has related the importance of the use of cross-linking agents in some studies, in the mean time, there are no studies comparing cross-linked or non-cross-linked chitosan-based membranes, as well as the deacetylation degree relation in the membranes’ biodegradation process, as mentioned by Paradossi *et al*. (1992) [27]. In the Paradossi *et al*. study. an 85% deacetylation degree is taken as a standard pattern, and higher degrees lower the biopolymer degradation, and lower degrees accelerate it. We can suggest that the results presented and compared to mentioned studies above, need more complex and ample evaluations to allow the chitosan-based membranes to effectively be used as an instrument for the tissue regeneration process.

## 5. Conclusions

There were variable degrees of inflammatory reaction, primary in the seven day experimental period. The chitosan-based membranes presented similar results to the collagen membranes, but the result showed were not satisfactory to indicate the use of the tested membranes clinically, suggesting the need for further studies. The molecular weight did not seem to affect the results of all groups in a relevant manner.
